# The development and evaluation of the Patient-Controlled Admission Fidelity Checklist

**DOI:** 10.3389/fpsyt.2026.1774052

**Published:** 2026-08-31

**Authors:** Maria Smitmanis Lyle, Anna Björkdahl, Clara Hellner, Sigrid Salomonsson, Alexander Rozental

**Affiliations:** 1 Centre for Psychiatry Research, Department of Clinical Neuroscience, Karolinska Institutet, & Stockholm Health Care Services, Region Stockholm, Stockholm, Sweden; 2 Department of Health, Education and Technology, Lulea Tekniska Universitet, Luleå, Sweden

**Keywords:** implementation fidelity, instrument development, mental health, Patient-Controlled Admission, psychiatric care

## Abstract

**Introduction:**

Patient-Controlled Admission (PCA) is an intervention aimed at increasing patient autonomy by allowing patients to admit themselves to psychiatric inpatient stays without physician assessment or staff gatekeeping. PCA is intended for patients with recurrent need of inpatient care, potentially those who would benefit from increased influence over the admission process. Studies suggest PCA may reduce involuntary care and improve patient empowerment. However, findings are difficult to interpret due to variation in implementation, lack of fidelity assessments, and the absence of an objective fidelity instrument.

**Aim:**

To develop and evaluate a fidelity measure for PCA capturing core standards of fidelity across clinical settings while remaining acceptable and user-friendly in routine practice.

**Methods:**

The development process of the fidelity instrument for chart review followed a five-step method: reviewing existing measures, analysing intervention components, drafting a fidelity checklist and guidelines, obtaining expert feedback, and piloting and refinement. Interrater reliability was assessed using Fleiss’ Kappa and percentage agreement, and acceptability and user-friendliness was evaluated in a routine clinical setting using Likert-scale questions.

**Results:**

The Patient-Controlled Admission Fidelity Checklist (PCA-FC) consists of three parts: A1 (PCA as part of the care plan), A2 (the PCA-agreement), and B (PCA inpatient care). Item-level percentage agreement ranged from 90% to 100%, with several items demonstrating complete agreement. Fleiss’ Kappa coefficients ranged from non-estimable variables for items with no response variability to perfect agreement (κ =1.00). The PCA-FC demonstrated strong acceptability and usability indicating that the checklist was perceived as easy to understand, complete, and apply in practice. Fidelity scores aggregated at the organisational level showed variation, with the lowest adherence observed in inpatient care items.

**Conclusion:**

The PCA-FC demonstrated high reliability for assessment of PCA fidelity through chart review. It may serve as a useful tool for implementation monitoring, clinical quality assurance, and research on effectiveness of PCA in psychiatric settings. However, fidelity is a multidimensional construct, and the PCA-FC represents an initial step in fidelity assessment that should be complemented by additional methods and further validation.

## 1 Introduction

Psychiatric inpatient care is an important component of mental healthcare, aiming to provide patients with a safe and supportive environment to receive care and treatment (1). However, there is a growing concern about low levels of patient engagement and participation in healthcare (2–4), with limited involvement being linked to poorer outcomes (5). Psychiatric care has been criticised for its paternalistic approach, where healthcare professionals, particularly physicians, predominantly control decisions regarding admission, care, treatment, and discharge (4, 6). In recent decades, there has been an growing emphasis on patient participation in psychiatric care (7). However, individuals seeking acute inpatient admission during periods of severe psychiatric distress frequently report feeling invalidated or dismissed during the assessment process. Healthcare staff may judge their symptoms as insufficiently severe to justify admission, regardless of the patient’s subjective experience of crisis (8). Being denied care despite perceived need may contribute to feelings of rejection and disempowerment, potentially leading patients to avoid seeking care in the future. For others, it may trigger a repetitive and deteriorating pattern in which care is repeatedly sought but denied. On the other hand, many individuals with severe mental illness never seek care despite clear clinical need, possibly due to structural barriers, stigma, or previous negative encounter with healthcare (9).

Patient-Controlled Admission (PCA) has been described as a novel and promising approach, allowing patients to exert power over the admission process to psychiatric inpatient care, without being questioned by healthcare professionals (10). Studies on the effects of PCA have been conducted in various countries, including Sweden. Findings from these studies indicate a significant reduction of both the total numbers of days spent in inpatient care and the incidence of involuntary admissions (10–12). Additional findings suggest that PCA enhances patient autonomy, responsibilities, and confidence in management of daily life (13–17). Although two randomised controlled trials have been conducted (11, 18), the majority of existing studies on PCA rely on small-scale designs, often limited to single patient groups such as individual’s who self-harm or patients with schizophrenia spectrum disorders. This makes it harder to generalise findings or to understand the impact of PCA on the observed effects (19).

PCA is a collaborative care model included in the care plan which allows patients to admit themselves to psychiatric inpatient care. It involves a formalised written agreement between the patient, the outpatient care unit responsible for the patient’s ongoing care, and the inpatient ward where historically the patient has often been admitted. The PCA-agreement is an integral part of the patient’s crisis plan, including strategies for managing health deterioration, the duration of inpatient care under the PCA, and the patient’s preferences for interaction with healthcare staff during their inpatient stay (20). PCA should be offered to patients with the greatest need of inpatient care and to those who will benefit the most from its use, e.g. patients with a history of frequent admissions and substantial use of inpatient care (20). In Sweden, PCA is currently offered in 18 out of 21 regions, but the practice differs between the regions, and sometimes even within the same region (21). Although PCA is widely offered and has been positively received by patients, actual utilisation by self-admitting to inpatient care appears to be relatively low, with only about half of the patients with access to PCA ever using it and roughly one quarter doing so less than once per year (22). Consequently, two populations may be considered exposed to PCA: all patients with a PCA-agreement and therefore access to PCA, and the smaller group patients that uses it by self-admitting to inpatient care. In Region Stockholm, Sweden, PCA has been implemented and evaluated on a small-scale basis in three separate projects since 2014 for specific patient diagnostic groups: schizophrenia spectrum disorders (12), borderline personality disorders (16, 23–25), and eating disorders (17, 26, 27). In 2019, the Stockholm Regional Council decided that PCA should be available to more patients with severe mental health disorders. Therefore, a generic model (i.e., similar to all patients) with the same procedure for PCA is being implemented in all psychiatric clinics in Region Stockholm (20).

When interventions are implemented in healthcare settings, it is common for them to over time become diluted or locally adjusted resulting in the loss of key components and reduced effectiveness. This phenomenon often occurs in complex systems where multiple demands and limited resources challenge fidelity (28). Fidelity is defined as the extent to which an intervention is delivered as intended by its original developers (28–32), and is essential in the determination of whether an intervention achieves its desired effects (33–35). In absence of fidelity assessments in ongoing evaluations, it remains unclear whether the effects, or lack of effects, are explained by the intervention itself or by insufficient implementation. An illustration of this can be found in the INTERCARE study conducted in Switzerland. This nurse-led intervention aimed to reduce unplanned hospitalisations among nursing home residents through the implementation of six core components, including advance care planning and structured interprofessional communication. The study found that sites with high fidelity to the intervention protocol, as measured by a fidelity questionnaire of INTERCARE’s core components, experienced significantly greater reductions in unplanned hospital admissions compared to those with lower fidelity (36). This demonstrates that implementation quality is critical to achieve intended effects, even when it involves a well-designed intervention. Furthermore, it is essential to recognize that positive outcomes may be observed even when an intervention is not performed according to its intended protocol. This variability may stem from factors such as differences in training and experience, as well as contextual and organisational factors such as the therapeutic relationships between patients and healthcare staff (30, 37).

In psychiatric care, fidelity instruments are essential for safeguarding both quality of care and patient safety. For instance, the use of fidelity measures, in Assertive Community Treatment (ACT) has been linked to better symptom management, fewer hospitalisations, and improved functioning among individuals with severe mental illness (38). Another example is the First Episode Psychosis Services Fidelity Scale (FEPS-FS) which has provided a reliable means to assess the degree to which mental health teams deliver specialised, evidence-based care to individuals experiencing a first episode of psychosis (39). These tools help ensure that critical components are delivered consistently, thereby reducing risks and enhancing patient recovery (38, 39).

Despite growing interest in PCA, there is currently a lack of knowledge regarding how well PCA is implemented in routine care and the quality of its delivery. To our knowledge, the only fidelity measure available for self-admissions in 2026 is the Brief Admission Skåne Fidelity Measure (BASFM) (40). However, the Brief Admission model differs significantly from the generic model of PCA, and the BASFM primarily consists of subjective measures, such as assessing the tone of the conversation and level of engagement, which presents challenges for objective evaluation. These subjective assessments focus more on the quality of interpersonal relations, reflecting one dimension of fidelity. In contrast, fidelity measures based on chart review typically evaluate adherence to structural and procedural components, capturing another dimension of fidelity. The BASFM is conducted through video recordings that are subsequently rated, which can limit scalability and feasibility in large patient volumes. In contrast, fidelity measures based on chart review offer advantages in terms of efficiency and the ability to assess larger samples. Such instruments may also serve as valuable complements to observational measures like the BASFM, providing a more comprehensive evaluation of fidelity across different dimensions. Understanding variation in current practice is essential for both interpreting existing findings and guiding future development and evaluation efforts, and mapping variation in current practice can provide insights into how PCA is implemented across settings.

Evaluating implementation fidelity requires considering not only protocol adherence, but also the practicality and user-friendliness of tools and procedures. If tools are perceived as inconvenient or impractical, they may hinder consistent use and compromise the fidelity assessment process (28). The overall aim of this study was to develop and evaluate a new fidelity measure for PCA that captures core, minimum standards of fidelity to the PCA model that should be consistently met across all clinical settings, while also being acceptable and user-friendly for routine clinical use. The instrument is intended to be used both in routine clinical settings for internal evaluation, e.g. unit managers and quality and improvement professionals, and in future research studies requiring standardized and reliable measurement of fidelity to PCA, e.g. investigating the relationship between effects and fidelity.

This study aimed to address the following research questions:

1. What would be the structure and content of a reliable and valid fidelity instrument for PCA?
2. What is the interrater reliability of the newly developed fidelity instrument?
3. How acceptable and user-friendly is the fidelity instrument perceived by those who apply it?
4. To what extent is there variation in fidelity scores when the instrument is applied in current practice?

## 2 Methods

### 2.1 Study design

To ensure a systematic development of the fidelity instrument, we followed the five-step method outlined by Walton et al. (2020). This approach involves: (1) Reviewing previous measures; (2) Analysing intervention components; (3) Developing fidelity checklist and coding guidelines; (4) Obtaining feedback about the content; and (5) Piloting and refining (41). The study was conducted in a psychiatric healthcare setting in Stockholm, Sweden where PCA have been implemented and part of routine care. Data collection took place between April 2023 and November 2024.

#### 2.1.1 Step one: reviewing previous measures

During the first step we reviewed other fidelity checklists to inform the selection of components for the PCA fidelity measure. The only fidelity checklist available for PCA was the Brief Admission Skåne Fidelity Measure (BASFM) (42). However, the Region Stockholm PCA model differed significantly from the Brief Admission Skåne model, and the BASFM primarily consisted of subjective measures, which posted challenges in objective evaluation. As an alternative, we examined fidelity measures familiar to us, including the Acceptance and Commitment Therapy Fidelity Measure (ACT-FM) (43), Fidelity of Rehabilitation (FiRe) (44), and the Promoting Independence in Dementia (PRIDE) fidelity checklist (41). The existing fidelity instruments were reviewed to gain inspiration regarding the structure, item formulation, and scoring methods for fidelity measurement. Other examples of fidelity measures are the Antipsychotic Medication Management Fidelity Scale (45), the Physical Health Care Fidelity Scale (46), and scales to evaluate quality of medication management (47).

#### 2.1.2 Step two: analysing intervention components

In the second step, we systematically analysed key components specific to the generic model of PCA to define which elements should be assessed to determine fidelity. These were extracted from the regional guidelines on PCA manual used in Region Stockholm (available on request) by the first author who contributed to the development of the guidelines.

The regional guidelines on PCA defines the mandatory content of PCA and how it should be documented. In Region Stockholm, the care plan is an interdisciplinary document developed in outpatient care that describes the patient’s overall care needs, resources, goals, and planned treatment and interventions. It is regularly reviewed and should be formally evaluated at least annually. Importantly, integrating PCA into the care plan is itself a key component of the model, as it ensures that PCA is incorporated into the patient’s broader treatment strategy and agreed upon within the care team. In contrast, the PCA agreement specifies the practical conditions for PCA. The PCA-agreement is documented in the crisis plan template and must include conditions for PCA use, length of stay, inpatient ward including contact details for the ward, guidance on what to do if the bed is not available, suggested activities during PCA inpatient care, which approach from healthcare staff the patient desires, and when to seek care in other ways than using PCA. The crisis plan is a patient-held document that the patient keeps and can refer to when in need. When having a PCA-agreement, patients can initiate short inpatient admissions without prior clinical assessment by contacting the inpatient ward directly. Admissions are conducted by an authorized nurse and documented in a designated PCA admission template in the patient medical record. At admission, risk assessment and individualised care planning for the specific inpatient stay are performed and documented in collaboration with the patient. Standard PCA episodes last 1–4 days and may be used up to three times per month, however, individual adaptations may be made. If significant clinical deterioration, elevated risk, or need for prolonged care is identified during the PCA inpatient stay, PCA may be converted to ordinary voluntary or involuntary inpatient care following physician assessment. Discharge is conducted by an authorized nurse, documented using a designated PCA template and includes an evaluation of the admission and a final risk assessment. As part of the PCA implementation, nurses responsible for PCA admissions and discharge receive specific training in suicide risk assessment, which is necessary for the delegated responsibility to admit and discharge patients without a physician assessment.

Each section of the manual was independently reviewed by multiple team members working with the implementation of PCA in Region Stockholm. The purpose of the review was to identify and operationalise the predefined PCA-components described in the regional guidelines into measurable checklist items. Discrepancies in interpretation were discussed until agreement was reached. The process was used to ensure consistency with the regional PCA guidelines rather than to establish expert consensus regarding the content of PCA. These identified components were then categorized into two distinct groups: A) Patients with a PCA-agreement and B) Patients who have used their PCA-agreement for admission to inpatient care.

#### 2.1.3 Step three: developing fidelity checklists

The development process led to the creation of the Patient-Controlled Admission Fidelity Checklist (PCA-FC), consisting of three parts for chart review, each part corresponding to a different aspect of the PCA intervention: A1 PCA as a part of the care plan, A2 The PCA-agreement, and B PCA inpatient care, presented in **Table 1**. The three checklist parts assess different aspects of PCA documented within the patient’s medical record. Part A1 and A2 apply to all patients with access to PCA, whereas part B applies only when a PCA admission has occurred. Each part contains a defined number of items (A1: 6 items, A2: 9 items, B: 8 items) with binary response options (“Yes” or “No”) to assess the presence of PCA components documented in patient medical records and reduce the need for subjective interpretation.

**Table 1:** The Patient-Controlled Admission Fidelity Checklist (PCA-FC).

Checklist part	Patient category
A1 PCA as part of the care plan	For all patients with access to PCA
A2 The PCA-agreement	For all patients with access to PCA
B PCA inpatient care	For all patients who have used PCA inpatient care

Additionally, distinct guidelines were formulated for chart review by professionals to provide detailed instructions on how to effectively utilise the checklist. The reason to base the instrument on chart review was guided by the structure of PCA implementation itself. Regional guidelines clearly state that key elements of the intervention, such as inclusion of PCA as part of the care plan, documentation of the PCA-agreement, and details of each admission, must be consistently recorded in the patient’s medical record. Thus, clinical documentation not only reflects clinical practice but also serves as a concrete indicator of fidelity, making chart review a suitable and practical method for assessment.

#### 2.1.4 Step four: obtaining feedback

To evaluate the content validity of the checklist, a panel of six experts was assembled through existing professional networks, consisting of individuals with specialized knowledge about PCA, healthcare providers with hands-on experience in PCA, patient-safety experts, inpatient ward managers with no prior experience of PCA, and patient representatives with lived experience as patients. This diverse expert group reviewed the checklist and the guidelines to ensure that the items were relevant and comprehensive. Feedback was obtained from all panel members, and based on their input, the checklist and guidelines were subsequently revised based on their input. These revisions included the addition of illustrative examples to the guidelines, adding checklist items, and rephrasing of checklist items to enhance clarity and reduce potential misunderstandings.

#### 2.1.5 Step five: piloting and refining checklists and guidelines

Finally, the instrument was piloted studied in two phases: (1) a standardised assessment using the same de-identified medical record extracts for all raters to evaluate interrater reliability; and (2) application of the checklist in the medical record system to evaluate acceptability and user-friendliness under routine clinical settings. A comparison of the two pilot phases is presented in **Table 2**. Participants were recruited from five adult psychiatric clinics, and one child and adolescent psychiatric clinic within Region Stockholm and the Centre for Psychiatry Research who were responsible for the implementation and evaluation of PCA within Region Stockholm.

**Table 2:** Overview of the objectives, procedures, and outcomes of the two pilot phases in the development and evaluation of the PCA-FC.

Feature	Phase 1	Phase 2
Purpose	Interrater reliability	Feasibility, acceptability and usability
Assessment setting	Standardised	Routine clinical practice
Source material	Same de-identified PCA documentation extracts for all raters	PCA documentation identified by each rater in the electronic medical record system
Records assessed	Same records by all raters	Different records reviewed by each rater
Main outcome	Agreement between raters	Fidelity results and user experience

In the first phase of the pilot test, we extracted, and de-identified 60 random patient medical record entries related to PCA from the Region Stockholm’s electronic patient medical record system, TakeCare: 20 care plan templates, 20 crisis plan templates, and 20 PCA inpatient care templates. The patients included in the entries were individuals with a PCA-agreement from various clinics within Region Stockholm, encompassing diagnoses such as schizophrenia spectrum disorders, borderline personality disorders, bipolar disorders, and eating disorders. All participant received the written guidelines with instructions on how to complete the PCA-FC. No formal training was provided, as the checklist is intended to be used in routine practice without prior training. A total of 20 PCA checklists, corresponding to 60 individual checklist parts, were completed by eight participants individually, presented in **Table 3**, distributed as follows: 20 for A1 PCA as part of the care plan, 20 for A2 The PCA Agreement, and 20 for B PCA inpatient care, aimed at assessing interrater reliability. The checklists were completed using the Research Electronic Data Capture (REDcap) system, a secure web-based application designed for data collection and management in research settings. Participants accessed the de-identified patient medical record entries stored within a secure folder and systematically reviewed. All eight participants rated each checklist item within the REDcap platform.

**Table 3:** Participants pilot test, phase 1.

Participant	Experience in working with PCA	Profession
A	No	Medical secretary
B	Yes	Implementation and Research support
C	Yes	Implementation and Research support
D	No	Patient safety coordinator
E	No	Safety coordinator
F	No	Patient representative
G	Yes	Implementation and Research support
H	Yes	Implementation and Research support

In the second phase of the pilot test, nine participants, presented in **Table 4**, completed a total of 101 checklist parts by direct reviewing patient medical record entries in TakeCare, using a paper and pen checklist format. Although local documentation templates may vary, standardised PCA-related formulations have been implemented across Region Stockholm. This provides a common basis for identifying and assessing PCA components during chart review. The patient medical record entries reviewed were linked to the care plan template, crisis plan template and inpatient PCA-specific templates, making them easy to locate within the electronic patient medical record system. As a result, the text volume was relatively small. The distribution included 37 checklist parts related to A1 PCA as part of the care plan, 37 for A2 The PCA-agreement, and 27 for B PCA inpatient care. To assess the acceptability and practicality of the checklist parts, we designed a set of questions that focused on key aspects of the rating process. Participants timed the duration of the checklist completion themselves and responded to three Likert-scale questions (rated from 1 = very easy to 7 = very hard) addressing: (1) Understanding how to complete the checklist; (2) Completing the checklist; and (3) Locating the necessary information to complete the checklist after each task. Although these questions were not formally validated in the context of this study, they were chosen to capture participants experiences related to aspects of usability. Additionally, participants were given the opportunity to provide additional comments at the end of completing the checklist parts.

**Table 4:** Participants pilot test, phase 2.

Participant	Experience in working with PCA	Profession
1	Yes	Inpatient ward manager
2	Yes	Outpatient unit manager
3	Yes	Inpatient ward nurse
4	Yes	Outpatient unit nurse
5	No	Patient representative
6	No	Patient representative
7	No	Patient representative
8	No	Medical secretary
9	No	Patient safety coordinator

No revisions were made to the included items, as they were based on the mandatory content specified in the guidelines on PCA.

### 2.2 Data analysis

Data from the completed checklists were extracted from REDCap and compiled into a Microsoft Excel sheet by the first author, including both the checklists and paper survey results. Interrater reliability was assessed using Fleiss’ Kappa (κ) to evaluate agreement among eight raters (48), using R statistical software (version 4.4.2). In Phase 1, all raters independently assessed the same 20 de-identified observations for each checklist item. Percentage agreement was calculated for each item as the proportion of ratings in agreement among all ratings across the 20 observations per item. Fleiss’ Kappa was calculated separately for each checklist item based on the ratings of the eight raters across the 20 observations. For items where all ratings fell within a single response category (i.e., complete agreement and no variance in ratings), Fleiss’ Kappa could not be estimated and was therefore reported as not applicable. While Fleiss’ Kappa is commonly used to assess interrater reliability by adjusting for chance agreement, the binary nature of our checklist items (yes/no), combined with a high level of observed agreement and a skewed distribution of responses resulted in unstable and uninformative Kappa values. Therefore, percentage agreement was used additionally as a more transparent and interpretable measure of interrater reliability in this study (49). Reliability analyses were conducted using data from pilot test Phase 1, as all raters assessed the same record extracts.

To analyse the acceptability and user-friendliness of the fidelity instrument, descriptive statistics were calculated for each item based on responses from the participants. Mean (M) and standard deviation (SD) were computed using R statistical software (version 4.4.2). Descriptive fidelity results were derived from pilot test Phase 2, in which participants applied the checklist to records identified in routine clinical practice.

### 2.3 Ethical considerations

This study was approved by the Swedish Ethical Review Authority (Etikprövningsmyndigheten), approval number 2020-06498. An amendment to the original application was also submitted and approved (2023-06430-02).

## 3 Results

The PCA-FC constitutes a single fidelity checklist assessing adherence to predefined minimum standards of PCA comprising three components: A1 PCA as part of the care plan containing six items; A2 The PCA-agreement containing nine items; and B PCA inpatient care containing eight items (see **Supplementary Files 1**, **2**). Thus, the components reflect different aspects of the same model rather than three separte fidelity scales. Each item represents a component considered essential according to the PCA guidelines and was therefore assessed individually rather than weighted or combined into a composite score. The final included items for assessment are presented for each checklist part in **Table 5**.

**Table 5:** Included items for each checklist part of the PCA-FC.

A1 PCA as part of the care plan	A2 The PCA-agreement	B PCA inpatient care
PCA documented in care plan	Conditions for PCA use	Admission process by authorized staff
Start date of PCA specified	Max days per PCA stay	Assessment of suicide risk at admission
End date of PCA specified	Max PCA stays/month	Justification for suicide risk at admission
Ward specified	Ward specified	Patient’s goals documented
Max days per PCA stay	Contact number for ward	Individual care plan for PCA stay
Max PCA stays/month	Guidance if bed is unavailable	Discharge process by authorized staff
	Suggested activities during PCA stay	Assessment of suicide risk at discharge
	Staff approach desired	Justification for suicide risk at discharge
	When to seek other care	

### 3.1 Interrater reliability

Percentage agreement was consistently high across checklist items and generally exceeded the corresponding Fleiss’ Kappa values. Item-level percentage agreement ranged from 90% to 100%, whereas Fleiss’ Kappa coefficients ranged from -0.006 to 1.000. According to McHugh’s interpretation of Kappa statistics (49), the observed agreement ranged from no agreement to almost perfect agreement. Several items showed very high percentage agreement but comparatively low Kappa values due to the highly skewed distribution of responses. For five items in part A1, all raters provided identical ratings across all 20 repeated observations, resulting in 100% agreement and insufficient variability to estimate Fleiss’ Kappa. Among the remaining items, agreement ranged from moderate to strong, with the highest agreement observed for item 7 in checklist part A2 (κ = 1.000). A small number of items showed lower agreement than the remainder of the checklist. Inspection of the rating patterns indicated that this was largely attributed to disagreement involving a single rater. Detailed reliability results are presented in **Table 6**.

**Table 6:** Item-level interrater reliability assessed by percentage agreement and Fleiss’ Kappa (κ) based on Phase 1 pilot testing using de-identified medical record extracts (n = 20 observations per item rated by 8 raters).

Checklist part	Item	Percentage agreement (%)	Fleiss’ κ
A1	1	100%	–
	2	100%	–
	3	100%	–
	4	97%	0.027
	5	100%	–
	6	100%	–
A2	1	94%	0.462
	2	99%	0.926
	3	99%	0.893
	4	99%	-0.006
	5	99%	0.851
	6	96%	0.755
	7	100%	1.000
	8	93%	0.714
	9	98%	0.750
B	1	96%	0.774
	2	99%	0.949
	3	94%	0.756
	4	90%	0.368
	5	99%	0.935
	6	95%	0.434
	7	99%	0.925
	8	96%	0.840

Fleiss’ κ could not be estimated for items A1.1, A1.2, A1.3, A1.5, and A1.6 because all ratings fell within a single response category.

### 3.2 Acceptability and user-friendliness

The mean time to complete checklist parts A1 and A2 (completed jointly) was 3.95 minutes (SD=2.4) while checklist part B took an average of 5.52 minutes (SD=3.4). Responses to the three Likert-scale questions on acceptability and practicality are summarized with their respective means and standard deviations in **Table 7**.

**Table 7:** Responses to questions addressing acceptability and user-friendliness based on Phase 2 pilot testing in routine clinical practice by 9 raters.

Question	Parts A1 and A2 M(SD)	Part B M(SD)
Understanding how to complete the checklist	1.38 (0.9)	1.37 (0.6)
Completing the checklist	1.23 (0.8)	1.26 (0.4)
Locating the necessary information to complete the checklist	1.5 (1.4)	1.9 (1.2)

Responses to questions, rated from 1 = very easy to 7 = very hard.

Three participants provided comments after completing the checklist parts; two reported that the checklist became easier to use after the first administration, and one participant provided a comment regarding their individual results.

### 3.3 Fidelity results

**Tables 8**–**10** present the descriptive statistics for each item in the fidelity checklist. The unit of analysis was the individual patient record. Because the PCA-FC was developed as a checklist of minimum fidelity requirements, results are reported at the item level as the percentage of records meeting the criterion specified in the question (i.e., marked “Yes”). No composite fidelity score was calculated. To provide an overall indication of adherence, the proportion of records fulfilling all applicable items within each checklist part was calculated and is presented in **Table 11**.

**Table 8:** Descriptive statistics for fidelity items of checklist A1 PCA as part of the care plan based on Phase 2 pilot testing in routine clinical practice by 9 raters.

Item	No. Pat. records	Yes (n/N)	Percent yes (%)
1	37	32/37	86.5
2	37	32/37	86.5
3	37	30/37	81.1
4	37	32/37	86.5
5	37	31/37	83.8
6	37	31/37	83.8

**Table 9:** Descriptive statistics for fidelity items of checklist A2 the PCA-agreement based on Phase 2 pilot testing in routine clinical practice by 9 raters.

Item	No. Pat. record	Yes (n/N)	Percent yes (%)
1	37	35/37	94.6
2	37	32/37	86.5
3	37	32/37	86.5
4	37	34/37	91.9
5	37	34/37	91.9
6	37	32/37	86.5
7	37	31/37	83.8
8	37	29/37	78.4
9	37	22/37	59.5

**Table 10:** Descriptive statistics for fidelity items of checklist B PCA inpatient care based on Phase 2 pilot testing in routine clinical practice by 9 raters.

Item	No. Pat. record	Yes (n/N)	Percent yes (%)
1	27	22/27	81.5
2	27	23/27	85.2
3	27	19/27	70.4
4	27	20/27	74.1
5	27	19/27	70.4
6*	21	16/21	76.2
7*	21	17/21	81.0
8*	21	13/21	62.0

*If admissions change from PCA to voluntary or non-voluntary care,items 6, 7, and 8 should not be completed because the relevant PCA procedures were no longer part of the inpatient care episode.

**Table 11:** Proportion of patient records fulfilling all applicable items within each PCA-FC part.

Checklist part	No. records	Fulfilling all applicable items n (%)
A1	37	30 (81.1%)
A2	37	18 (48.6%)
B	27	7 (26.0%)

Adherence was generally high across most items. However, lower adherence was observed for part A2 The PCA-agreement item A2.9 (59.5%). Within Part B PCA inpatient care, adherence was lowest for Items B.3 (70.4%), B.4 (74.1%), B.5 (70.4%) and B.8 (62.0%), indicating greater variation in the fulfilment of these PCA components compared with the remaining items.

Complete adherence was observed in 30 of 37 records for part A1, 18 of 37 records for part A2, and 7 of 27 records for part B. These findings suggest that although adherence to individual items was generally high, fulfilment of all applicable PCA components was less common, particularly for PCA inpatient care.

## 4 Discussion

In this study, we developed the Patient-Controlled Admission Fidelity Checklist (PCA-FC), a structured instrument designed to assess fidelity to PCA through chart review, which demonstrated high interrater reliability. Furthermore, participants reported that completing the checklists was quick, with an average time of 4 in minutes for checklist parts A1 and A2, and 5.5 in minutes for part B. They also found the checklist easy to understand, complete, and navigate for locating necessary information. This suggests that the design of the checklists was user-friendly and efficient, which likely contributed to the high levels of interrater reliability.

There are no directly comparable fidelity instruments that measure PCA to our knowledge. This limits the ability to benchmark our results against existing tools. However, the process undertaken in developing and testing the PCA-FC aligns with established practices in implementation science (38). Although a formal consensus procedure such as Delphi (50) was not used, the PCA-FC was developed directly from the predefined components described in the regional PCA guidelines. Consequently, expert review focused on interpretation and operationalization of these components rather than determining the content of PCA. Although several items likely reflect underlying principles of PCA our study did not seek to explicitly identify or validate the core conceptual principles underpinning PCA. Future conceptual work may help strengthen the theoretical foundation of fidelity assessment in PCA. However, its relatively general content allows for application across diverse organizational contexts. The conceptual breadth makes it suitable for use with different PCA designs, target groups, and levels of implementation maturity. Several items reflect practical prerequisites for accessing and using PCA. For example, the inclusion of ward contact information may not directly assess patient autonomy or other self-admission behaviour, but it supports accessibility and the patient’s ability to independently initiate admission when needed.

In this study, interrater reliability was assessed using both Fleiss’ Kappa and percentage agreement. The findings indicate differences in interrater reliability across the checklist. Notably, five of the six items in part A1 demonstrated 100% agreement among raters. For these items, Fleiss’ Kappa could not be estimated because all ratings fell within a single response category. In contrast, item 4 in part A1 showed a percentage agreement of 97% but a Fleiss’ Kappa of 0.027, illustrating how Kappa may underestimate agreement when response distributions are highly skewed (51). Consequently, percentage agreement was considered an important complementary measure of interrater reliability in this study. Using percentage agreement aligns with practices in other research, such as Liddy et al. (2011), who also employed percent agreement to evaluate interrater reliability in primary care medical record abstraction. Their study reported an overall percentage agreement of 94.3%, indicating strong reliability (52). The high percentage agreement observed across the three PCA-FC parts indicates that the assessment process in our study demonstrated high interrater reliability. The presence of some items with lower percentage agreement, attributed to a single participant, suggest that occasional deviations may occur due to individual differences. While it is uncertain why this deviation occurred, this could be explained by external factors. This participant’s data may have been influenced by factors such as misunderstanding of the checklist items, interruption during completion, or other external variables. Future studies may require additional observational data to determine the underlying explanation. Replication of the testing with different raters could help to confirm whether these variations are indeed due to individual differences.

The separation of checklist components applicable to all patients with a PCA-agreement, i.e. access to self-admit, and those applicable only to patients who have used PCA by self-admitting reflects both the structure of the model and a potential analytic opportunity. Future studies may examine whether patterns of adherence in the care plan and PCA-agreement components are associated with subsequent utilization of PCA, thereby providing additional insights into the implementation and functioning of PCA.

The fidelity results revealed considerable variation, ranging from 59.5% to 94.6% adherence across different components of PCA, with the lowest adherence observed in items related to inpatient care documentation. This variability suggests that while some components were implemented with high fidelity, others encountered challenges potentially compromising their delivery. Contributing factors may include differences in training resource availability, and contextual adaptations (28, 53). The variation in fidelity scores across items also suggests that the instrument has the capacity to discriminate between components of PCA. This indicates that the PCA-FC captures relevant differences in implementation fidelity and may be useful in identifying specific areas needing improvement. Providing short training in documentation practices and the application of the PCA-FC may increase the likelihood of both improved documentation quality and more consistent fidelity to the PCA protocol.

The development of the PCA-FC for quality assessment, although focused on a limited part of the patient medical record, may offer a new perspective on how chart review and documentation practices can be approached more broadly. By operationalizing fidelity to a specific care model, such as PCA, the instrument encourages systematic reflection on what constitutes essential information in clinical documentation. This structured approach not only facilitates targeted quality improvement efforts but may also inspire broader shifts in documentation efforts, emphasizing completeness and clinical relevance. In this way, the PCA-FC has the potential to complement existing quality assurance tools and serve as an inspiration for the development of similar tools for other parts of patient medical records, thereby supporting a more structured documentation practice and ultimately contributing to safer, high-quality care. The PCA-FC may also contribute to broader quality assurance efforts by supporting adherence to general documentation standards in psychiatric care. Moreover, implementation studies have demonstrated that chart review in general, can lead to meaningful improvements in documentation quality (54).

Several fidelity instruments have been developed through structured expert consultation and consensus procedures, often combining empirical evidence, clinical expertise, and stakeholder input to establish content validity (55, 56). In contrast, the PCA-FC was derived from predefined PCA components specified in the regional guidelines, with expert review focusing primarily on operationalization and interpretation of these components. The PCA-FC was developed to capture minimum standards of fidelity to the PCA model that should be consistently met across all clinical settings. However, fidelity is a multidimensional construct (57), and the PCA-FC does not capture all possible aspects of adherence to PCA, e.g. relational components such as communication between outpatient and inpatient units or introducing PCA to new staff. Current guidelines on fidelity assessment commonly recommend triangulation, i.e. combining data from different sources to enhance validity (57). The PCA-FC relies solely on chart review and to capture other dimensions, complementary methods such as observations, interviews, or patient-reported experiences are needed. Several fidelity assessment frameworks combine information from multiple sources, including documentation review, interviews, observations, and stakeholder reports, to capture different dimensions of implementation fidelity (56, 58). Therefore, the PCA-FC should be viewed as a first step in the development of a broader fidelity assessment framework for PCA. The PCA-FC focuses on documented fidelity and should therefore be regarded as one component of a broader fidelity assessment strategy. As with other fidelity assessments based on chart review, including the Antipsychotic Medication Management Fidelity Scale, the Physical Health Care Scale, and medication management quality scales (45–47), the findings are dependent on the completeness and accuracy of clinical documentation. Missing or undocumented information was treated as nonadherence, which may underestimate fidelity when PCA-related activities have been performed but not recorded. However, an important strength of our study is that PCA documentation is recorded using dedicated templates containing standardized PCA-related content. In addition, standardized wording has been implemented across Region Stockholm, which supports consistent documentation and reduces the risk that relevant information is difficult to locate. Nevertheless, chart review cannot fully capture all dimensions of fidelity, highlighting the importance of complementary assessment methods such as observations, interviews, and patient-reported measures. However, accurate and comprehensive medical documentation is essential for ensuring high-quality care and supporting clinical decision making (59).

The PCA-FC was developed as a checklist of minimum standards rather than a scale intended to generate an overall fidelity score. Consequently, adherence was examined at the item level, as a composite score could obscure deficiencies in individual components considered essential to PCA. The realistic goal should be to achieve 100% “yes” responses on all items, reflecting a minimum degree of fidelity to PCA. Consistent with this rationale, the proportion of records fulfilling all applicable items was also examined. While complete adherence was common for part A1, substantially fewer medical records fulfilled all criteria in parts A2 and B. This illustrates how relatively high adherence to individual items may coexist with incomplete adherence to all minimum requirements. Any deviations from 100% “yes” responses on all items necessitates a thorough analysis to identify the underlying causes of missing elements from the patient medical records, as incomplete documentation may have implications for both clinical quality and patient safety (60). Such deviations may reflect deficiencies in documentation, implementation challenger, or other contextual factors. However, our study was not designed to determine reasons for non-adherence, and no conclusions regarding causality can therefore be drawn. Future studies could examine whether alternative summary indices provide meaningful and valid representation of overall PCA fidelity, while retaining information about adherence to individual minimum requirements.

Items 2, 3, 7, and 8 in checklist part B PCA inpatient care, which relate to suicide risk assessment during admission and discharge, showed percent yes responses of 85.2%, 70.4%, 81.0%, and 62.0% respectively. In Sweden, national guidelines mandate the documentation of suicide risk assessments within healthcare settings. According to the Swedish National Board of Health and Welfare, suicide risk assessments must be thoroughly documented to ensure patient safety and continuity of care. These guidelines are part of the broader framework of national recommendations aimed at improving the quality and consistency of healthcare services (61). Additionally, these items were included because admission with PCA involves a modified admission procedure in which aspects of the initial assessments are performed by nursing staff rather than physicians. Consequently, suicide risk assessment constitutes both an important patient safety procedure and a key operational component of PCA inpatient care. Although suicide risk assessments remain a standard component in many clinical protocols, there is a growing consensus in the literature that their predictive validity is limited. Several systematic reviews and meta-analysis have demonstrated that structured risk assessments have poor sensitivity and specificity in identifying individuals who will go on to engage in suicidal behaviour (62–64). Nonetheless, suicide risk assessment was included in the PCA-FC in recognition of its continued role in current guidelines. This decision reflects a pragmatic alignment with the existing healthcare standards rather than an endorsement of the assessment’s predictive utility. As the field shifts towards more dynamic, individualised, and preventative approaches to suicide prevention, emphasising ongoing monitoring and systemic factors, it is likely that the role of static risk assessment will become less central. Future versions of the PCA-FC should remain adaptable to such developments, potentially rephrasing suicide risk assessment with more holistic formulations.

It is important that fidelity instruments like this are accompanied by mechanisms for interpretation, feedback, and subsequent action incorporating both quantitative outcomes from the PCA-FC and qualitative insights derived from reviewing patient medical records and other sources such as on-site observations and interviews with patients and staff. Evidence indicates that audit and feedback interventions are most impactful when they establish a feedback loop that integrates these quantitative and qualitative data sources (65).

Existing literature indicate that when interventions are implemented with high fidelity, patients tend to report more favorable outcomes, including satisfaction, engagement, and perceived quality of care (33, 66). Prior research on PCA has shown high levels of patient satisfaction (13–17), however, there remains a lack of empirical assessment regarding fidelity to PCA. To advance understanding in this domain, future studies should integrate fidelity evaluations alongside assessment of other outcomes.

### 4.1 Limitations

Although the PCA-FC was developed through a systematic process, including testing and feedback from experts with clinical and implementation expertise in healthcare and PCA, several limitations should be considered when interpreting the results of this study. Although patient perspectives were represented through patient representatives with lived experience, patients receiving care were not recruited specifically in the development process. Involvement of a broader patient sample may have contributed additional perspectives on the relevance of the checklist.

There is currently no established gold standard for assessing fidelity to PCA, nor clear criteria for what constitutes an adequate level of fidelity. This makes it difficult to benchmark or validate the results against a known reference.

Although the checklist was developed directly from the predefined PCA components described in the regional guidelines, and therefore did not require a formal consensus process to determine PCA content, a structured method such as a Delphi process involving clinicians, researchers, and patients could have provided a more systematic evaluation of content validity (50). While the PCA-FC was derived from guideline-defined PCA components rather than an explicitly articulated theoretical framework of PCA principles, the relationship between these components and broader conceptual principles of PCA has not been formally examined. Future studies could investigate whether the current items adequately represent the underlying principles of the PCA model.

The checklist uses a binary format, which facilitates assessment of the presence or absence of predefined PCA components and reduces the need for subjective interpretation by raters. However, it does not capture the quality or depth of documentation. While this was a deliberate design choice to enhance usability and scalability, assessing quality would likely require more extensive training and introduce subjectivity into the rating process.

Another limitation is that the fidelity assessment was exclusively based on documentation in PCA-specific templates within the patient medical record. Therefore, the results reflect documented PCA practice rather than actual care delivery. If PCA activities were performed but not documented within the designated templates, they would not be captured by the assessment.

The study did not control for participant’s training effects. Those with greater familiarity or interest in PCA may have found the checklist easier to apply, and the possibility of self-selection bias cannot be excluded, as participants were recruited through professional networks.

Finally, although the PCA-FC was well received and found feasible in a pilot context, the instrument capturing user experience was not validated, and routine clinical environments are often characterized by time pressure and competing interests. These factors may influence whether the tool is used consistently and as intended in everyday practice.

### 4.2 Conclusions

This study represents an important first step in the development of a fidelity checklist for PCA. The PCA-FC demonstrated high interrater reliability, strong acceptability, and user-friendliness in clinical settings, indicating that it is both a feasible and practical tool for assessing adherence to key components of PCA as documented in the patient medical record. By enabling systematic assessment of how PCA is documented and delivered, the checklist can support implementation monitoring, promote adherence to key components of the intervention, and contribute to quality improvement efforts. The PCA-FC also facilitates identification of variation in practice, which is important to understand when interpreting clinical outcomes and evaluating the overall effectiveness of PCA. However, fidelity is a multidimensional construct, and future research should complement chart review-based assessments with additional methods capable of capturing aspects of implementation that extend beyond of documentation, such as patient- and professional experiences and to what extent PCA-principles are enacted in practice. Further validation is needed, including assessment of predictive validity, e.g. by examining whether higher fidelity scores are associated with improved patient experiences, person-cantered processes, or clinical outcomes. Another aspect could be to investigate how the fidelity results using the PCA-FC are affected by training and increased work experience of PCA. Such work would represent an important next step in the development of a comprehensible fidelity assessment for PCA.

## Data Availability

The raw data supporting the conclusions of this article will be made available by the authors, without undue reservation.
