# Supplementary material for: The development and evaluation of the Patient-Controlled Admission Fidelity Checklist
Source: Front Psychiatry. 2026 Aug 31;17:1774052. doi: 10.3389/fpsyt.2026.1774052 (PMC13572607; doi:10.3389/fpsyt.2026.1774052)
Supplement: Supplementary file 1 [file SupplementaryFile1.docx]

**Instructions Patient-Controlled Admission Fidelity Checklist (PCA-FC)**

**Instructions A1. PCA as part of the care plan**
Open the journal template *Individual Plan* or *Care and Crisis Plan*, depending on which template is used in the unit, and control that the keyword *Access to Patient-Controlled Admission* is included.

**1. It is documented that the patient has access to PCA in the patient’s care plan – Yes/No.**
For yes, it must be stated that the patient has access to PCA in the care plan.

**2.** **The care plan includes information on the start date of PCA (date from.) – Yes/No.**
For yes, the start date must be stated.

**3. The care plan includes information on the end date of PCA (date to) – Yes/No.**
For yes, the end date must be stated.

**4. The care plan includes information on which inpatient ward PCA can be used – Yes/No.**
For yes, the ward must be specified.

**5. The care plan includes information on the number of days per PCA admission the patient can use – Yes/No.**
For yes, number of days must be specified.

**6. The care plan includes information on the number of PCA admissions per month the patient can use – Yes/No.**
For yes, number of admissions must be specified.


**Instructions A2. The PCA Agreement as Part of the Crisis Plan**
Open the journal template *Crisis Plan* or *Care and Crisis Plan*.

**1. The PCA agreement includes information about when the patient may use PCA – Yes/No.**
For yes, it must be stated when the patient may use PCA, or reference early warning signs of deterioration documented in the crisis plan.

**2. The PCA agreement includes information on the number of days per PCA admission the patient can use – Yes/No.**
For yes, number of days must be specified.

**3. The PCA agreement includes information on the number of PCA admissions per month the patient can use – Yes/No.**
For yes, number of admissions must be specified.

**4. The PCA agreement includes information on which inpatient ward PCA can be used – Yes/No.**
For yes, the ward must be specified.

**5. The PCA agreement includes contact telephone number(s) for the inpatient ward – Yes/No.**
For yes, the phone number must be stated.

**6. The PCA agreement includes information on what the patient can do if the bed for PCA is temporarily occupied – Yes/No.**
For yes, options the patient can use in this scenario must be stated.

**7. The PCA agreement includes information on activities the patient can engage in during PCA inpatient stay – Yes/No.**
For yes, at least one activity must be documented. This can include both staff-led activities and things the patient can do themselves. Examples: “Daily Talks. Sensory room. Walks. Sudoku.”

**8. The PCA agreement includes information on the desired approach or attitude of healthcare staff during PCA inpatient stay – Yes/No.**
For yes, it must be stated how the patient wishes staff to relate to/support/interact with them during the inpatient care period. Example: “Help/motivation to get up for breakfast and spend time in the common room during the day.”

**9. The PCA agreement includes information on when the patient should seek care by other means than PCA – Yes/No.**
For yes, this must be stated.

**Instructions Checklist B. PCA Inpatient Care**

Open the journal templates *Admission, Patient-Controlled Admission*; *Individual plan*; *Discharge note, nurse – Patient-Controlled Admission* / *Discharge note, physician*.

**1. Admission was conducted by a nurse with delegation *or* a physician– Yes/No.**
Review *Admission, Patient-Controlled Admission* and control who was responsible. Ensure they have valid delegation according to signed delegation form on the date the documentation was made.
For yes, the one responsible for the admission is either a nurse with valid delegation or a physician.

**2. Suicide risk assessment is documented at admission – Yes/No.**
Review *Admission, Patient-Controlled Admission* and control the documentation under the keyword *Suicide risk assessment*.
For yes, one of the four risk levels must be filled in: Minimal risk, Some risk, High risk, Very high risk.

**3. Justification for the suicide risk assessment is documented at admission – Yes/No.**
Review *Admission, Patient-Controlled Admission* and control the documentation under the keyword *Suicide risk assessment*.
For yes, a free-text justification must be provided under the keyword *Justification suicide risk assessment*.

**4. There is information about what the patient wishes to receive help with during the admission – Yes/No.**
For yes, this must be stated.

**5. An individual care plan for the PCA inpatient stay has been established – Yes/No.**
For yes, the *Individual plan* template must be present.

*If the care period has shifted from PCA to extended care under ordinary regulations (HSL or LPT), stop here.*

**6. Discharge was conducted by a nurse with delegation *or* a physician – Yes/No.**
Review *Discharge note, nurse – Patient-Controlled Admission* and control who was responsible. Ensure they have valid delegation according to signed delegation form on the date the documentation was made.
For yes, the one responsible for discharge is either a nurse with valid delegation or a physician (using *Discharge note, physician*).

**7. Suicide risk assessment is documented at discharge – Yes/No.**
Review *Discharge note, nurse – Patient-Controlled Admission* or *Discharge note, physician* and control the documentation under the keyword *Suicide risk assessment*.
For yes, one of the four risk levels must be filled in: Minimal risk, Some risk, High risk, Very high risk.

**8. Justification for the suicide risk assessment is documented at discharge – Yes/No.**
Review *Discharge note, nurse – Patient-Controlled Admission* or *Discharge note, physician* and control the documentation under the keyword *Suicide risk assessment*.
For yes, a free-text justification must be provided under keyword *Justification suicide risk assessment*.
