# Supplementary material for: The development and evaluation of the Patient-Controlled Admission Fidelity Checklist
Source: Front Psychiatry. 2026 Aug 31;17:1774052. doi: 10.3389/fpsyt.2026.1774052 (PMC13572607; doi:10.3389/fpsyt.2026.1774052)
Supplement: Supplementary file 2 [file SupplementaryFile2.docx]

# **Patient-Controlled Admission Fidelity Checklist (PCA-FC)**

The PCA-FC consists of three parts. This checklist is designed to ensure fidelity to the Patient-Controlled Admission (PCA) method and are completed through chart review. For each part of the checklist, there are accompanying guidelines with instructions for each item.

| **Part** | **Patient Category** |
| --- | --- |
| A1. PCA as part of the care plan. | For all patients with access to PCA. |
| A2. The PCA-Agreement. | For all patients with access to PCA. |
| B. PCA Inpatient care. | For all patients who have had PCA Inpatient care. |

**A1. PCA as part of the care plan**

|  | **Question** | **Yes** | **No** | **Comments** |
| --- | --- | --- | --- | --- |
| 1. | It is documented that the patient has access to PCA in the patient’s care plan. |  |  |  |
| 2. | The care plan includes information on the start date of PCA (date from.) |  |  |  |
| 3. | The care plan includes information on the end date of PCA (date to). |  |  |  |
| 4. | The care plan includes information on which inpatient ward PCA can be used. |  |  |  |
| 5. | The care plan includes information on the number of days per PCA admission the patient can use. |  |  |  |
| 6. | The care plan includes information on the number of PCA admissions per month the patient can use. |  |  |  |
|  | **Total number:** |  |  |  |

**A2. The PCA-Agreement as part of the Crisis Plan**

|  | **Question** | **Yes** | **No** | **Comments** |
| --- | --- | --- | --- | --- |
| 1. | The PCA-agreement includes information about when the patient may use PCA. |  |  |  |
| 2. | The PCA-agreement includes information on the number of days per PCA admission the patient can use. |  |  |  |
| 3. | The PCA-agreement includes information on the number of PCA-admissions per month the patient can use. |  |  |  |
| 4. | The PCA-agreement includes information on which inpatient ward PCA can be used. |  |  |  |
| 5. | The PCA-agreement includes contact telephone number(s) for the inpatient ward. |  |  |  |
| 6. | The PCA-agreement includes information on what the patient can do if the bed for PCA is temporarily occupied. |  |  |  |
| 7. | The PCA-agreement includes information on activities the patient can engage in during PCA inpatient stay. |  |  |  |
| 8. | The PCA-agreement includes information on the desired approach or attitude of healthcare staff during PCA inpatient stay. |  |  |  |
| 9. | The PCA-agreement includes information on when the patient should seek care by other means than PCA. |  |  |  |
|  | **Total number:** |  |  |  |

**B. PCA Inpatient Care.**

|  | **Question** | **Yes** | **No** | **Comments** |
| --- | --- | --- | --- | --- |
| 1. | Admission was conducted by a nurse with delegation ***or*** a physician. |  |  |  |
| 2. | Suicide risk assessment is documented at admission. |  |  |  |
| 3. | Justification for suicide risk assessment is documented at admission. |  |  |  |
| 4. | There is information about what the patient wishes to receive help with during the admission. |  |  |  |
| 5. | An individual care plan for the PCA inpatient stay has been established. |  |  |  |
| 6. | Discharge was conducted by a nurse with delegation ***or*** a physician* |  |  |  |
| 7. | Suicide risk assessment is documented at discharge* |  |  |  |
| 8. | Justification for suicide risk assessment is documented at discharge* |  |  |  |
| * | Provided that the PCA inpatient stay has not been converted from PCA to regular care according to the Health and Medical Services Act (HSL) or the Cumpulsory Psychiatic Act (LPT); if so, disregard questions 6-8. |  |  |  |
|  | **Total number:** |  |  |  |
